# Effects of Bariatric Endoscopy on Non-Alcoholic Fatty Liver Disease: A Comprehensive Systematic Review and Meta-Analysis

**DOI:** 10.3389/fendo.2022.931519

**Published:** 2022-06-17

**Authors:** Mengting Ren, Xinxin Zhou, Yunyun Zhang, Feifei Mo, Jinpu Yang, Mosang Yu, Feng Ji

**Affiliations:** Department of Gastroenterology, The First Affiliated Hospital, Zhejiang University School of Medicine, Hangzhou, China

**Keywords:** endoscopy, non-alcoholic fatty liver disease (NAFLD), bariatrics, liver function tests, meta-analysis

## Abstract

**Background and objective:**

Endoscopic bariatric and metabolic therapies (EBMTs) are emerging minimally invasive therapeutic options for obesity and its related complications, including non-alcoholic fatty liver disease (NAFLD). This study aimed to evaluate the effects of EBMTs on NALFD in patients with obesity.

**Methods:**

Four databases were searched until Nov 2021. Randomized controlled trials (RCTs) and observational studies reporting liver-related outcomes following Food and Drug Administration (FDA)-approved and non-FDA-approved EBMTs were included. Liver parameters, metabolic parameters, and weight loss were evaluated. Risk of bias was assessed using the “risk of bias” tool in the Cochrane Collaboration for RCTs and the Methodological Index for Non-Randomized Studies criteria for observational studies.

**Results:**

Thirty-three studies with 1710 individuals were included. Regarding the effects of EBMTs on liver fibrosis, a significant decline of NAFLD Fibrosis Score, but not transient elastography-detected liver stiffness or Fibrosis-4 Index, was observed. EBMTs significantly improved liver steatosis (control attenuation parameter and Hepatic Steatosis Index), NAFLD Activity Score, and Homeostasis Model Assessment of Insulin Resistance. EBMTs reduced serum levels of alanine transaminase, aspartate aminotransferase, and gamma-glutamyl transpeptidase considerably. Moreover, EBMTs had reducing effects on the serum levels of triglycerides and total cholesterol as well as body weight.

**Conclusions:**

Our meta-analysis suggested that EBMTs could ameliorate NAFLD based on the evidence of improved liver steatosis, liver function, and insulin resistance. Large-scale, prospective, long-term studies are warranted to clarify the role of EBMTs in patients with different stages of NAFLD.

## 1 Introduction

Non-alcoholic fatty liver disease (NAFLD) is the most common cause of chronic liver disease worldwide, affecting up to 25% of the general population (1, 2). Furthermore, ~25% of NAFLD patients can develop non-alcoholic steatohepatitis (NASH), which may progress to fibrosis and cirrhosis (3). Obesity and insulin resistance represent the most important risk factors for NAFLD development (1). Although some emerging drugs such as sodium-glucose cotransporter 2 (SGLT2) inhibitors have shown benefits for NAFLD (4), approved pharmacotherapy for NAFLD is lacking. The backbone of NAFLD management is lifestyle modification through diet and exercise (5). Unfortunately, lifestyle interventions rarely permit total body weight loss (TBWL) of 7–10% to improve steatosis and liver biochemistry, or TBWL >10% to improve fibrosis (6). Recent studies have shown the benefit of bariatric surgery on the histologic and serologic features of NAFLD/NASH (7). Nevertheless, as with any surgical procedure, bariatric surgery is expensive and associated with potential health risks, especially in patients with end-stage liver disease. Therefore, novel, safe, and efficacious therapies for NAFLD/NASH are needed urgently.

In recent years, endoscopic bariatric and metabolic therapies (EBMTs) have emerged as less invasive methods targeted to achieve comparable results to bariatric surgery for obesity and its metabolic comorbidities at a more affordable cost and a lower risk of complications (8). Currently, six bariatric and metabolic-endoscopy devices have been approved by the US Food and Drug Administration (FDA): Orbera Intragastric Balloon System (IGB; Apollo Endosurgery, Austin, TX, USA), Obalon Balloon System (Obalon Therapeutics, Carlsbad, CA, USA), transpyloric shuttle (TPS; BAROnova, San Carlos, CA, USA), OverStitch Endoscopic Suturing System (Apollo Endosurgery) for endoscopic sleeve gastroplasty (ESG), incisionless operating platform (IOP; USGI Medical, San Clemente, CA, USA) for primary obesity surgery endoluminal (POSE), and AspireAssist (Aspire Bariatrics, King of Prussia, PA, USA) for aspiration therapy (AT). Some EBMTs are currently in clinical trials, including other types of IGB, such as the Spatz Adjustable Balloon System and ReShape Duo Balloon, and small intestinal EBMTs, including duodenal jejunal bypass liner (DJBL), duodenal mucosal resurfacing (DMR), and incisionless magnetic anastomosis system (IMAS). In addition to the effects of significant reduction in body weight (8), an increasing number of studies have reported EBMTs to have positive effects on NAFLD, including liver function, steatosis, and fibrosis (9–11). However, the small sample size limited the statistical robustness of the results. Besides, most of the studies evaluated only a few NAFLD biomarkers.

We undertook a systematic review and meta-analysis for comprehensive assessment of the therapeutic effects of EBMTs for NAFLD. We explored the potential role of EBMTs (IGB, ESG, POSE, AT, DJBL, and DMR) for NAFLD therapy by evaluating liver fibrosis, steatosis, NAFLD Activity Score (NAS), liver volume and liver function. Changes in insulin resistance, lipid profile, and weight loss were also reported.

## 2 Methods

### 2.1 Protocol and Guidance

This study was carried out in accordance with Preferred Reporting Items for Systematic Reviews and Meta-Analyses guideline (12). The study protocol is registered with PROSPERO (CRD42021247382).

### 2.2 Search Strategy

Searches were developed and conducted by an experienced research librarian and lead author (MR) in the databases MEDLINE, EMBASE, Web of Science, and Cochrane Central Register of Controlled Trials (CENTRAL) from inception to Nov 2021. We also reviewed the references of identified trials for additional relevant literature. Unpublished “gray” literature and ongoing trials were also searched. The full search strategy is presented in **Supplementary Appendix 1**.

### 2.3 Eligibility Criteria and Study Selection

Randomized controlled trials (RCTs) and observational studies aiming to evaluate EBMTs in obese/NAFLD/type-2 diabetes mellitus (T2DM) patients which reported liver-related outcomes (e.g., liver fibrosis, steatosis, and liver function) were eligible for inclusion. Eight types of bariatric and metabolic endoscopy were included with no restriction on language: IGB, TPS, ESG, POSE, AT, DJBL, DMR, and IMAS. Included studies were required to report outcomes at ≥3 months and be undertaken for patients ≥18 years of age. For comparative studies, relevant data were selected for inclusion.

The exclusion criteria were: (i) review articles, case reports/series, editorials, and comments; (ii) patients with cirrhosis intending to undergo liver transplantation; (iii) studies with fewer than five participants. In case of overlap between articles reporting on the same cohort, we included the study with the largest cohort or the most recent study.

Retrieved studies were imported into Endnote™ X9 (Clarative Analytics, Philadelphia, PA, USA), and duplicate hits were deleted. Two researchers (MR and XZ) screened articles independently by title and abstract. Subsequently, potentially eligible studies were checked in full text for inclusion by the same authors. Disagreements were solved by consensus.

### 2.4 Data Abstraction

Two independent reviewers (MR and JY) extracted data from each study using a predesigned form for data extraction. The data we sought were: (i) study characteristics (first author, year of publication, study design, country); (ii) characteristics of study participants at baseline (disease type, total number of participants, age, sex, body mass index [BMI]); (iii) intervention characteristics (intervention type, follow-up duration); (iv) clinical outcomes.

### 2.5 Assessment of Study Quality

RCTs were assessed for methodologic quality using the “risk of bias” tool within CENTRAL (13). Two independent reviewers (MR and MY) assessed each study for risk of bias. The overall risk of bias was considered to be “high” if ≥1 domain was rated as a high risk of bias. A RCT was considered to have a “low” risk of bias if all domains were considered to be at low risk. Otherwise, the trial was considered as having an “unclear” risk of bias. For observational studies, we assessed the risk of bias using Methodological Index for Non-Randomized Studies (MINORS) criteria (14). Each domain was scored 0, 1, or 2, with a maximum sum of 16 for noncomparative studies, and 24 for comparative studies.

### 2.6 Data Synthesis and Statistical Analyses

The meta-analysis was undertaken using a random-effects model. Continuous outcomes were analyzed using the mean difference (MD) and associated 95% confidence intervals (CIs). Primary outcomes were liver fibrosis (transient elastography-detected liver stiffness, NAFLD Fibrosis Score [NFS], and Fibrosis-4 Index [FIB-4]), liver steatosis (control attenuation parameter [CAP] and Hepatic Steatosis Index [HSI]), NAS, liver volume, and level of liver enzymes (alanine transaminase [ALT], aspartate aminotransferase [AST], and gamma-glutamyl transpeptidase [GGT]). Secondary outcomes were Homeostasis Model Assessment of Insulin Resistance (HOMA-IR), weight loss (body weight, BMI, TBWL, excess weight loss [EWL]), as well as levels of glycated hemoglobin A1c (HbA1c), and the lipid profile (triglycerides [TG], total cholesterol [TC], high-density lipoprotein-cholesterol [HDL-C], low-density lipoprotein-cholesterol [LDL-C]).

We assessed clinical heterogeneity based on the protocol and methodology of the included studies. Statistical heterogeneity was investigated using the I^2^ test: I^2^ <25% denoted “low” heterogeneity; I^2^ = 25–50% denoted “moderate” heterogeneity; I^2^ >50% denoted “high” heterogeneity. To identify the potential source of bias, subgroup analysis and meta-regression analysis were carried out, if possible. Meta-regression analysis was performed under random effects model using the Restricted Maximum Likelihood (REML) method by including baseline BMI, TBWL, and duration of the follow-up as covariates. We also undertook sensitivity analysis by removing a specific study from the meta-analysis. Funnel plot and Egger’s regression asymmetry test were used to evaluate publication bias. Significance was established at a two-sided α-level of 0.05. Statistical analyses were carried out with Stata 16.0 (Stata Corporation, College Station, TX, USA).

## 3 Results

### 3.1 Study Selection

**Figure 1:**
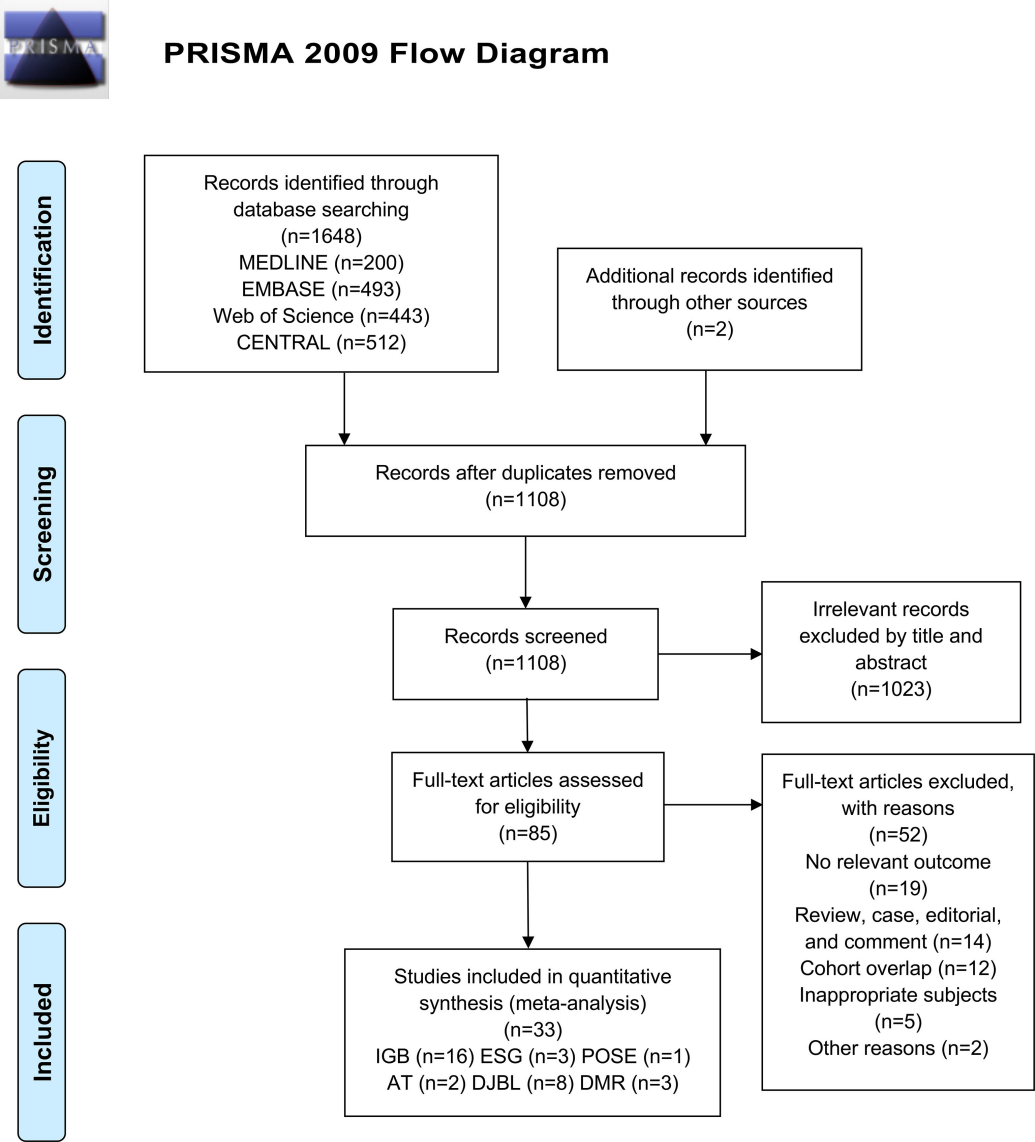
Flowchart detailing the search for studies based on PRISMA 2009.

Our search of databases identified 1648 records, of which 542 were duplicates. Two additional records were identified by a manual search. Among these articles, 1023 were excluded following screening of the title/abstract, and an additional 52 articles were excluded after full-text review. Finally, 33 studies remained eligible for this systematic review and meta-analysis, of which 26 were peer-reviewed published studies and seven were conference abstracts (**Figure 1**).

### 3.2 Study Characteristics

A total of 1710 participants were enrolled in the included studies. Study characteristics are summarized in **Table 1**. Sixteen studies evaluated IGB, among which one RCT (21) compared IGB *vs.* sham; three observational studies compared IGB *vs.* no IGB (22), cognitive-behavioral therapy (24), and lifestyle modification (25), respectively; the remaining 12 studies (9, 15–20, 23, 26–28, 44) were observational noncomparative studies. One prospective observational study (29) compared ESG *vs.* IGB. Two observational noncomparative studies (10, 30) evaluated ESG. One prospective noncomparative study assessed POSE (31). Two RCTs (32, 33) compared AT *vs.* lifestyle counseling. Eight observational noncomparative studies (11, 34–40, 45, 46) evaluated DJBL. Two prospective noncomparative studies (41, 42) and one RCT (43) compared DMR *vs.* sham. With regard to TPS and IMAS, no study fulfilled the inclusion criteria.

**Table 1:** Characteristics of included studies.

Study	Intervention	Patient population	Country	Study design	Total subjects	Age	Gender(Female, %)	Baseline BMI (kg/m^2^)	Follow-up	Outcomes
Frutos et al., 2007 (15)	IGB	obese patients	Spain	prospective noncomparative	31	40.1 ± 11.1	21(67.7%)	55.2 ± 6.9	6 mo	liver volume, weight, BMI, EWL
Ricci et al., 2008 (16)	IGB	obese patients	Italy	retrospective noncomparative	103	41.3 ± 10.4	65 (63.1%)	42.1 ± 5.8	6 mo	ALT, GGT, HOMA-IR, BMI
Donadio et al., 2009 (17)	IGB	obese patients	Italy	prospective noncomparative	40	36.7 ± 10.6	29 (72.5%)	44.9 ± 8.9	6 mo	ALT, AST, GGT, HOMA-IR, HbA1c, weight, BMI, TG, TC, HDL-C
Forlano et al., 2010 (18)	IGB	obese patients	Italy	prospective noncomparative	120	38.6 ± 12	77 (59.2%)	43.1 ± 8	6 mo	ALT, GGT, HOMA-IR, weight, BMI, TG
Sekino et al., 2011 (19)	IGB	obese patients	Japan	retrospective noncomparative	8	39 ± ?	3 (37.5%)	41.6 ± 7.5	6 mo	ALT, AST, GGT, liver volume, HOMA-IR, HbA1c, weight, EWL, TG, HDL-C, LDL-C
Stimac et al., 2011 (20)	IGB	obese patients	Croatia	prospective noncomparative	171	39.2 ± 10.5	111 (65%)	44 ± 4.3	6 mo	ALT, GGT, weight, BMI, EWL, TG, TC, HDL-C, LDL-C
Lee et al., 2012 (21)	IGB	obese patients with NAFLD	Singapore	RCT (vs sham)	8	43 ± 14.6	5 (62.5%)	30.3 ± 3.1	6 mo	liver histology, BMI
Zerrweck et al., 2012 (22)	IGB	obese patients	France	retrospective comparative (vs no IGB)	23	44 ± 10.8	15 (65%)	65 ± 3.8	6 mo	ALT, GGT, HbA1c, weight, BMI, TC
Tai et al., 2013 (23)	IGB	obese patients	Taiwan	prospective noncomparative	28	31.5 ± 8.8	23 (82.1%)	32.4 ± 3.7	6 mo	ALT, AST, BMI, TG, TC, HDL-C, LDL-C
Majanovic et al., 2014 (24)	IGB	obese patients	Croatia	prospective comparative (vs cognitive-behavioral therapy)	60	38.6 ± 11.0	49 (81.7%)	38.6 ± 3.9	6 mo	ALT, GGT, weight, BMI, TG, TC, HDL-C, LDL-C
Takihata et al., 2014 (25)	IGB	obese patients	Japan	prospective comparative (vs lifestyle modification)	8	40.9 ± 13.9	3 (37.5%)	45.2 ± 5.9	6 mo	ALT, AST, GGT, liver volume, HOMA-IR, HbA1, weight, BMI, TG, HDL-C, LDL-C
Nguyen et al., 2017 (26)	IGB	obese patients with NAFLD	UK	retrospective noncomparative	135	47.1 ± 12.2	96 (71%)	41.7 ± 6.6	6 mo	ALT, AST, GGT, HOMA-IR, weight, BMI, TG, TC, HDL-C, LDL-C
Sarin et al., 2018 (24)	IGB	obese patients with NAFLD	India	prospective noncomparative	46	NR	NR	NR	NR	ALT, TE-LSM, weight, BMI
Bhakta et al., 2019 (27)	IGB	NR	US	retrospective noncomparative	12	NR	NR	NR	6 mo	ALT, TWL
Bazerbachi et al., 2021 (9)	IGB	obese patients with NAFLD	US	prospective noncomparative	21	54 ± 23.0	17 (81%)	43.2 ± 6.8	6 mo	ALT, AST, MRE, APRI, liver histology, HbA1c, weight, BMI, TC, LDL-C
Salomone et al., 2021 (28)	IGB	obese patients with NAFLD	Italy	retrospective noncomparative	26	53 ± 13.3	8 (31%)	35.1 ± 4.7	6 mo	ALT, AST, TE-CAP, TE-LSM, FIB-4, weight, TG, TC
Espinet-Coll et al., 2019 (29)	ESG	obese patients with NAFLD	Spain	prospective comparative (vs IGB)	15	47.0 ± 15.5	11 (73.3%)	39.8 ± 6.8	6 mo	ALT, AST, GGT, HSI, FLI, NFS, FIB-4, HbA1c, weight, BMI, TG, TC, HDL-C, LDL-C
Hajifathalian et al., 2020 (10)	ESG	obese patients with NAFLD	US	prospective noncomparative	118	46.7 ± 13	80 (68%)	40.0 ± 8.1	24 mo	ALT, AST, HSI, NFS, HOMA-IR, HbA1c, TWL
Reja et al., 2020 (30)	ESG	obese patients	US, Mexico	retrospective noncomparative	92	43.3 ± 11.4	NR	40.7 ± 7	3 mo	ALT, weight, BMI
Lopez-Nava et al., 2020 (31)	POSE	obese patients	Spain, US	prospective noncomparative	41	44.4 ± 9.4	25 (61%)	37.4 ± 1.7	6 mo	ALT, TE-CAP, TWL
Sullivan et al., 2013 (32)	AT	obese patients	US	RCT (vs lifestyle counseling)	10	38.7 ± 2.3	10 (100%)	42.0 ± 1.4	12 mo	ALT, AST, HbA1c, TWL, EWL, TG, TC, HDL-C, LDL-C
Thompson et al., 2016 (33)	AT	obese patients	US	RCT (vs lifestyle counseling)	111	42.4 ± 10.0	96 (86.5%)	42.0 ± 5.1	12 mo	ALT, AST, HbA1c, weight, TWL, EWL, TG, TC, HDL-C, LDL-C
de Jonge et al., 2013 (34)	DJBL	obese patients with T2DM	Netherlands	prospective noncomparative	17	51 ± 2	3 (17.6%)	37.0 ± 1.3	12 mo	ALT, AST, GGT, BMI, weight
Laubner et al., 2016 (35)	DJBL	obese patients with T2DM	Germany	prospective noncomparative	59	46 ± 9.8	NR	45.0 ± 7.3	12 mo	NFS, HbA1c, BMI, EWL
Stratmann et al., 2016 (36)	DJBL	obese patients with T2DM	Germany	prospective noncomparative	16	50.1 ± 7.9	3 (18.8%)	48.8 ± 8.5	12 mo	ALT, AST, GGT, HOMA-IR, HbA1c, weight, BMI, EWL, TG, HDL-C, LDL-C
Forner et al., 2017 (37)	DJBL	NR	Australia	combined retrospective and prospective noncompatative	114	51 ± 13	47 (41%)	41 ± 5.9	12 mo	ALT, AST, GGT, HbA1c, weight, BMI, TWL, TG, TC, HDL-C
Gollisch et al., 2017 (38)	DJBL	obese patients with T2DM	Germany	retrospective noncomparative	20	53.0 ± 10.2	14 (70%)	39 ± 6	12 mo	TE-CAP, TE-LSM, HbA1c, BMI
Karlas et al., 2018 (39)	DJBL	obese patients with T2DM	Germany	prospective noncomparative	31	57 ± ?	17 (59%)	39.5 ± 8.6	12 mo	ALT, GGT, TE-CAP, HbA1c, BMI
McMaster et al., 2019 (40)	DJBL	obese patients with T2DM	Australia	prospective noncomparative	19	range 20-63	14 (73.7%)	NR	12 mo	ALT, TWL, HDL-C
Ryder et al., 2019 (11)	DJBL	obese patients with T2DM	UK	prospective noncomparative	61	51.4 ± 7.2	28 (45.9%)	41.9 ± 7.4	12 mo	ALT, HbA1c, weight, BMI, TC, HDL-C
Haidry et al., 2019 (41)	DMR	patients with T2DM	Chile	prospective noncomparative	44	53.4 ± 7.5	16 (36%)	30.8 ± 3.5	24 w	ALT, AST, FIB-4, HbA1c, weight
van Baar et al., 2020 (42)	DMR	patients with T2DM	Netherlands, Belgium, Italy, UK, Chile	prospective noncomparative	46	55 ± 9.5	17 (37%)	31.6 ± 4.3	12 mo	ALT, HOMA-IR, HbA1c, weight
Mingrone et al., 2021 (43)	DMR	patients with T2DM	Belgium, Brazil, Italy, Netherlands, UK	RCT (vs sham)	56	58 ± 13.5	17 (30.4%)	31.5 ± 4.7	24 w	MRI-PDFF, HbA1c, weight, BMI

NR, not reported.

Studies were conducted in Europe (n = 16), north America (n = 6), Asia (n = 5), Australia (n = 2), and south America (n = 1); the remaining three international multicenter studies were conducted in Europe and America. When broken down by disease type, there were fifteen studies for obese patients, eight for obese T2DM patients, seven for obese NAFLD patients, and three for T2DM patients. The age and BMI at the time of the procedure varied from 31.5 years to 58 years and 30.3 kg/m^2^ to 65 kg/m^2^, respectively. The median duration of follow-up was 6 (range, 6–24) months.

### 3.3 Liver Fibrosis

**Figure 2:**
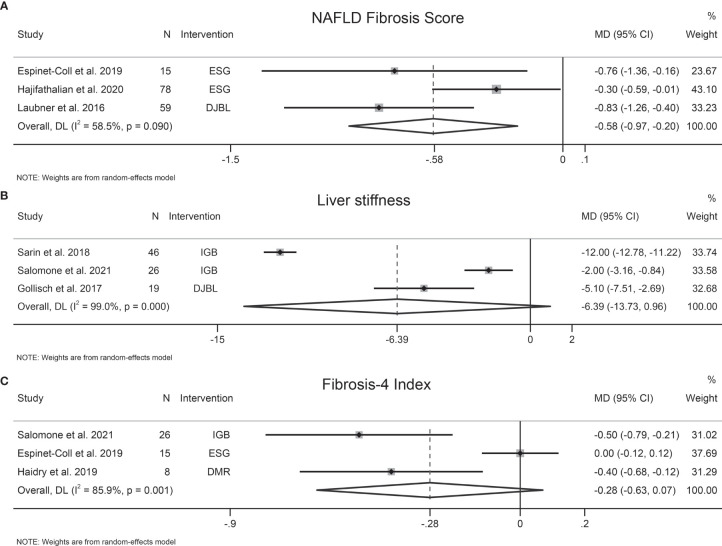
Forest plot of the effects of bariatric and metabolic endoscopy on liver fibrosis. **(A)** NAFLD Fibrosis Score (NFS), **(B)** transient elastography-detected liver stiffness, **(C)** Fibrosis-4 Index (FIB-4).

The effects of EBMTs on liver fibrosis were evaluated by transient elastography-detected liver stiffness (three studies, 91 participants), NFS (three studies, 152 participants), FIB-4 (three studies, 49 participants), and AST-to-platelet ratio index (APRI) (one study, 21 participants). EBMTs were associated with a significant decrease in NFS (MD −0.58 [95%CI −0.97 to −0.20]) (**Figure 2A**). Nevertheless, a non-significant difference was observed between EBMTs and transient elastography-detected liver stiffness (MD −6.39 kPa [95%CI −13.73 to 0.96]) (**Figure 2B**) or FIB-4 (MD −0.28 [95%CI −0.63 to 0.07]) (**Figure 2C**). One study (9) reported that IGB reduced APRI significantly by 0.73 (*P* = 0.005) and the magnetic resonance elastography-detected liver stiffness value by 0.3 kPa (*P* = 0.03).

Heterogeneity was high in terms of transient elastography-detected liver stiffness, NFS, and FIB-4, at I^2^ = 99%, 58.5%, and 85.9%, respectively. Sensitivity analysis showed that the results of our meta-analysis were stable. We did not evaluate publication bias due to the small number of trials.

### 3.4 Liver Steatosis

**Figure 3:**
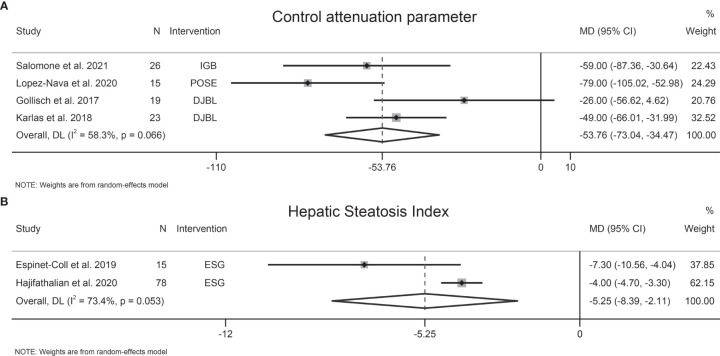
Forest plot of the effects of bariatric and metabolic endoscopy on liver steatosis. **(A)** control attenuation parameters (CAP), **(B)** Hepatic Steatosis Index (HSI).

The effects of EBMTs on liver steatosis were assessed by CAP (four studies, 83 participants), HSI (two studies, 93 participants), magnetic resonance imaging-proton density fat fraction (MRI-PDFF) (one study, 48 participants), and Fatty Liver Index (FLI) (one study, 15 participants). EBMTs were associated with a significant decrease in CAP (MD −53.76 dB/m [95%CI −73.04 to −34.47]) (**Figure 3A**) and HSI (MD −5.25 [95%CI −8.3 to −2.11]) (**Figure 3B**). However, Mingrone et al. found a non-significant relationship between DMR therapy and liver MRI-PDFF value (*P* = 0.096) (43). Similarly, another study showed that FLI did not change significantly following ESG (*P* = 0.280) (29).

Heterogeneity was high regarding CAP and HSI, at I^2^ = 58.3% and 73.4%, respectively. Sensitivity analysis showed that the results of our meta-analysis were stable. Publication bias was not assessed due to the small number of studies.

### 3.5 NAFLD Activity Score

**Figure 4:**
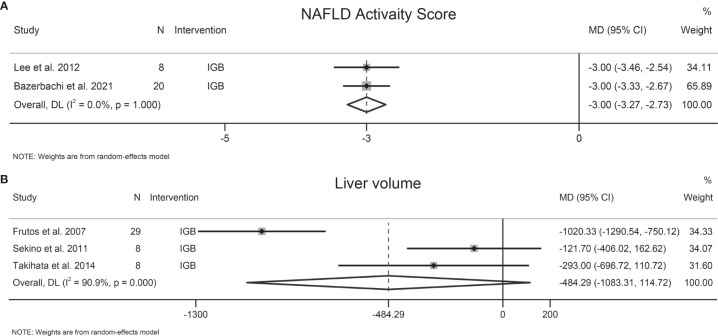
Forest plot of change in **(A)** NAFLD Activity Score (NAS) and **(B)** liver volume following bariatric and metabolic endoscopy.

Two studies involving 28 participants evaluated the effects of EBMTs on NAS, and both studies evaluated IGB. Following IGB, NAS was reduced significantly (MD −3 [95%CI −3.27 to −2.73]) (**Figure 4A**). Substantial heterogeneity was not found (I^2^ = 0.0%). The number of included studies was insufficient to carry out the Egger test.

### 3.6 Liver Volume

Three studies involving 45 patients explored the effect of EBMTs on liver volume, all of which evaluated IGB. Following IGB, a significant change of liver volume was not found (MD −484.29 cm^3^ [95%CI −1083.31 to 114.72]) (**Figure 4B**). Heterogeneity was high: I^2^ = 90.9%. Sensitivity analysis showed that the results of our meta-analysis were stable. A test of publication bias was not done due to the small number of trials.

### 3.7 Liver Enzymes

#### 3.7.1 ALT

**Figure 5:**
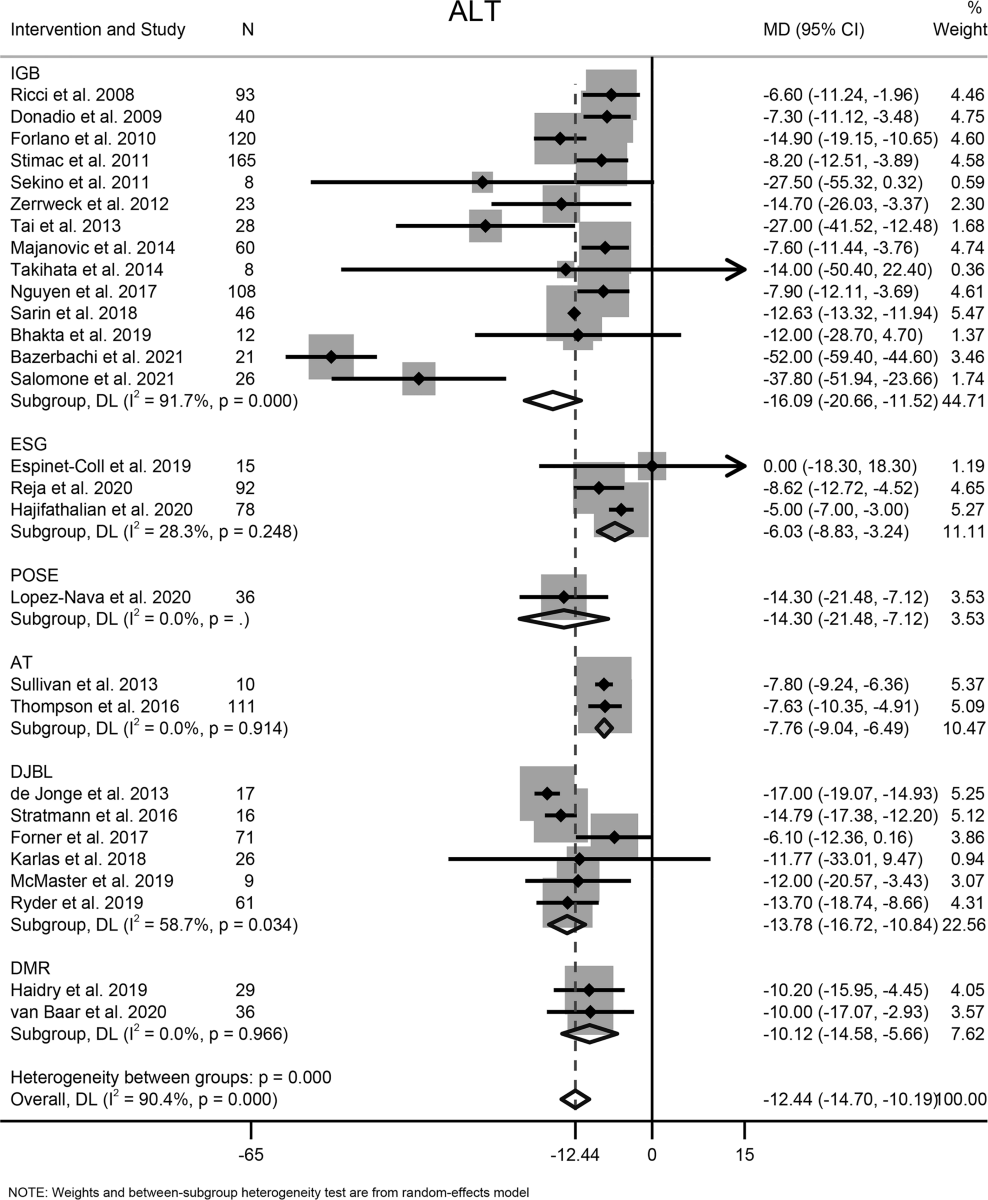
Forest plot of change in level of alanine transaminase following bariatric and metabolic endoscopy with subgroup analysis based on intervention type. ALT, alanine transaminase.

Twenty-eight studies involving 1365 participants reported the effects of EBMTs on the serum level of ALT. Overall, EBMTs were associated with significant reduction of the ALT level (MD −12.44 U/L [95%CI −14.70 to −10.19]) (**Figure 5**). Categorization into subgroups depending on intervention type revealed that all types of EBMTs could reduce the ALT level significantly (IGB: MD −16.09 U/L [95%CI −20.66 to −11.52], 14 studies, 758 participants; ESG: MD: −6.03 U/L [95%CI −8.83 to −3.24], three studies, 185 participants; AT: MD −7.76 U/L [95%CI −9.04 to −6.49], two studies, 121 participants; DJBL: MD −13.78 U/L [95%CI −16.72 to −10.84], six studies, 200 participants; DMR: MD −10.12 U/L [95%CI −14.58 to −5.66], two studies, 65 participants).

High heterogeneity among the studies was identified (I^2^ = 90.4%), so subgroup analysis, meta-regression, and sensitivity analysis were carried out. Subgroup analysis was conducted according to the intervention type, disease type, study design, BMI at baseline (<40, ≥40 kg/m^2^), and ALT level at baseline (<40, ≥40 and <60, ≥60 U/L). The ALT level at baseline was suspected of being a source of heterogeneity. On meta-regression analysis, the ALT level at baseline was a significant predictor for the magnitude of change in the ALT level (β = −0.66 [95%CI −0.80 to −0.51], *P* < 0.001). Sensitivity analysis demonstrated the stability of our meta-analysis. The funnel plot was symmetrical (**Figure 8A**) and Egger test *P* value was not significant (*P* = 0.762).

#### 3.7.2 AST

**Figure 6:**
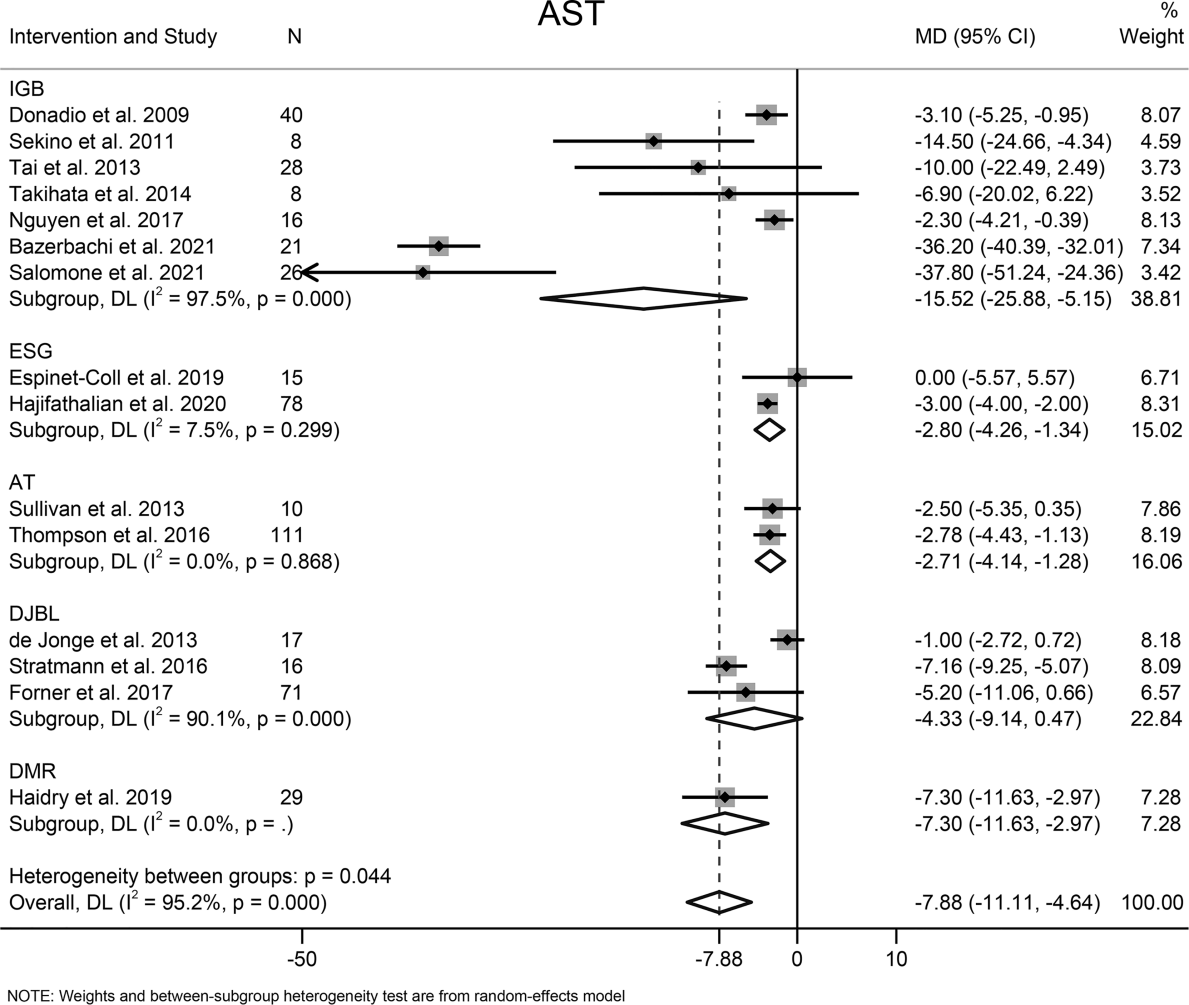
Forest plot of change in level of aspartate aminotransferase following bariatric and metabolic endoscopy with subgroup analysis based on intervention type. AST, aspartate aminotransferase.

The effects of EBMTs on the serum level of AST were assessed in 15 studies (494 participants). Overall, EBMTs significantly lowered the serum AST level (MD −7.88 U/L [95%CI −11.11 to −4.64]) (**Figure 6**). All types of EBMTs except DJBL (MD −4.33 U/L [95%CI −9.14 to 0.47], three studies, 104 participants) were associated with a reduction in the AST level (IGB: MD −15.52 U/L [95%CI −25.88 to 5.25], seven studies, 147 participants; ESG: MD −2.80 U/L [95%CI −4.26 to −1.34], two studies, 93 participants; AT: MD −2.71 U/L [95%CI −4.14 to −1.28], two studies, 121 participants).

The heterogeneity was high (I^2^ = 95.2%), so subgroup analysis, meta-regression analysis, and sensitivity analysis were conducted. Subgroup analysis was done according to the intervention type, disease type, study design, BMI at baseline (<40, ≥40 kg/m^2^), and AST level at baseline (<30, ≥30 and <60, ≥60 U/L). The AST level at baseline was suspected of being the source of heterogeneity. Further meta-regression analysis revealed that the AST level at baseline was a significant predictor for the magnitude of change in the AST level (β = −0.74, [95%CI −1.02 to −0.45], *P* < 0.001). Through sensitivity analysis, we found that the study by Bazerbachi and colleagues (9) was likely to be the main source of heterogeneity. After excluding that study, the heterogeneity decreased to 77.8%, and the mean difference in the AST level changed to −4.21 U/L [95%CI −5.86 to −2.56]. Though the funnel plot seemed to be visually asymmetrical (**Figure 8B**), no significant publication bias was detected by Egger test (*P* = 0.095).

#### 3.7.3 GGT

**Figure 7:**
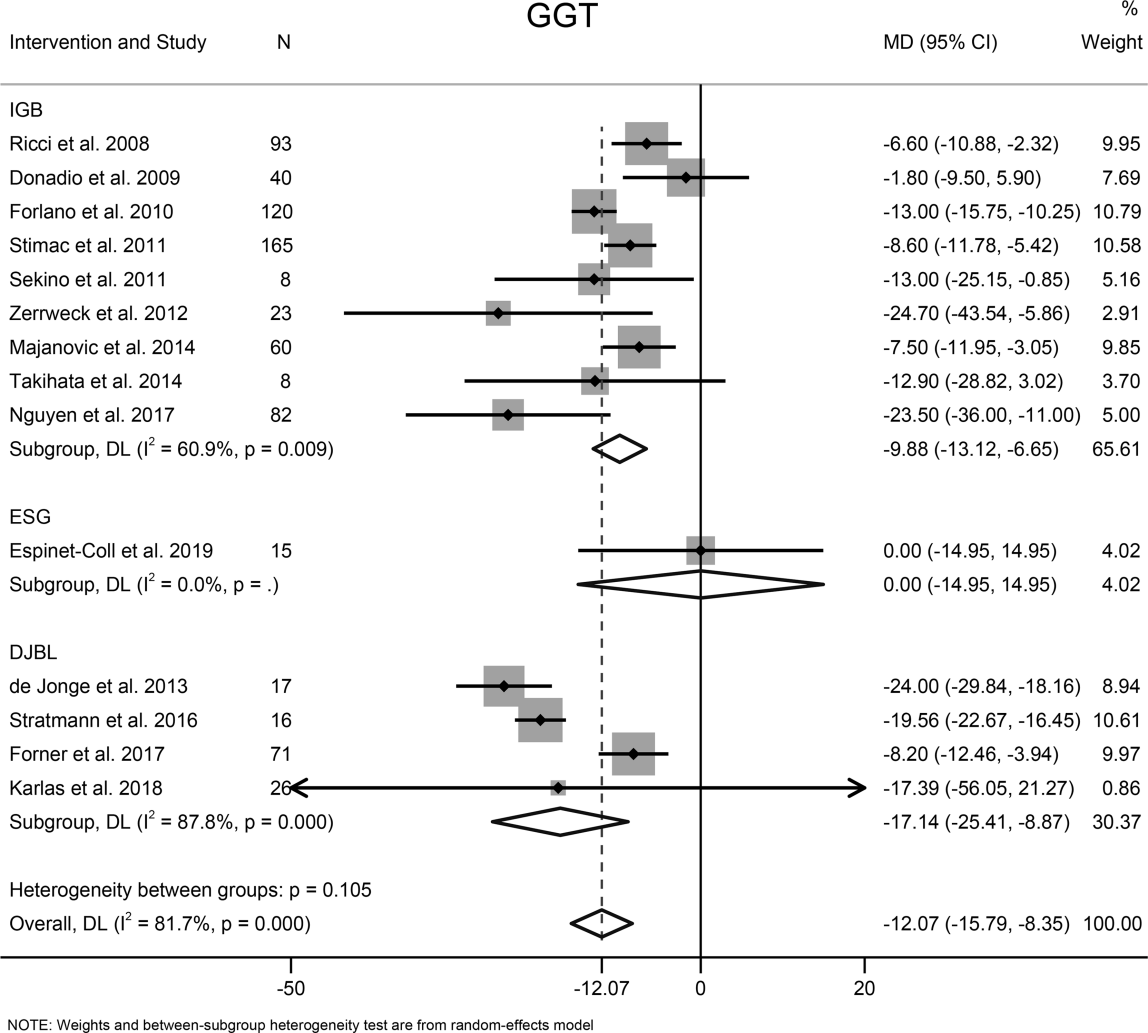
Forest plot of change in level of gamma-glutamyl transpeptidase following bariatric and metabolic endoscopy with subgroup analysis based on intervention type. GGT, gamma-glutamyl transpeptidase.

Fourteen studies with 744 participants evaluated the effects of EBMTs on the serum GGT level. Overall, bariatric metabolic endoscopy could reduce the serum level of GGT significantly (MD: −12.07 U/L [95%CI −15.79 to −8.35]) (**Figure 7**). IGB (MD −9.88 U/L [95%CI −13.12 to −6.65]) and DJBL (MD −17.14 U/L [95%CI: −25.41 to −8.87]) were associated with a significantly decreased GGT level.

**Figure 8:**
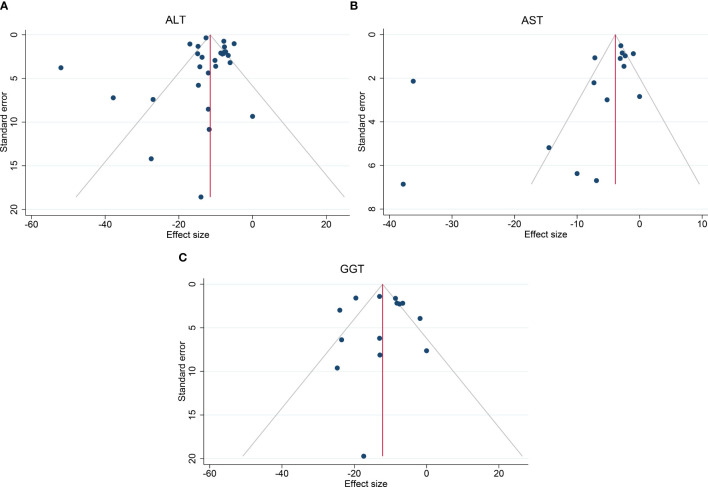
Funnel plots to assess publication bias. **(A)** Funnel plot of ALT, **(B)** Funnel plot of AST, **(C)** Funnel plot of GGT. ALT, alanine transaminase; AST, aspartate aminotransferase; GGT, gamma-glutamyl transpeptidase.

The heterogeneity test for the included studies revealed that the heterogeneity among the studies was high (I^2^ = 81.7%). Subgroup analysis was conducted according to the intervention type, disease type, study design, BMI at baseline (<40, ≥40 kg/m^2^), and GGT level at baseline (<35, ≥35 and <60, ≥60 U/L). The AST level at baseline was suspected of being a source of heterogeneity. Meta-regression analysis showed that the GGT level at baseline was a significant predictor for the magnitude of change in the GGT level (β = −0.47 [95%CI −0.70 to −0.25, *P* < 0.001). Sensitivity analysis suggested that the results were stable. The funnel plot was symmetrical (**Figure 8C**), and Egger test indicated no significant publication bias (*P* = 0.988).

### 3.8 Insulin Resistance

Nine studies involving 399 patients reported the effects of EBMTs on HOMA-IR. Following EBMTs, HOMA-IR was decreased significantly (MD −1.9 [95%CI −2.49 to −1.30]) (**Supplementary Figure 1A**), suggesting improved insulin resistance.

Nineteen studies involving 693 patients reported the effects of EBMTs on the HbA1c level, among which six-each evaluated IGB and DJBL, three evaluated DMR, and two-each evaluated ESG and AT. The HbA1c level was reduced significantly (MD −0.74 [95%CI −1.01 to −0.48]) (**Supplementary Figure 1B**) following EBMTs, which indicated improved glucose homeostasis.

### 3.9 Lipid Profile

Eighteen studies reported on the lipid profile in blood. EBMTs were associated with a significantly reduced serum level of TG (MD −0.33 mmol/L [95%CI −0.43 to −0.22], 14 studies, 709 participants) (**Supplementary Figure 2A**) and TC (MD −0.21 mmol/L [95%CI −0.32 to −0.09], 13 studies, 671 participants) (**Supplementary Figure 2B**). However, a significant change was not found in terms of the serum level of HDL-C (MD 0.03 mmol/L [95%CI −0.01 to 0.07], 14 studies, 582 participants) (**Supplementary Figure 2C**) or LDL-C (MD −0.08 mmol/L [95%CI −0.22 to 0.05], 11 studies, 483 participants) (**Supplementary Figure 2D**).

### 3.10 Weight Loss

All 33 studies reported weight loss-related outcomes. EBMTs reduced body weight (MD −12.18 kg [95%CI −15.42 to −8.93], 22 studies, 1224 participants) (**Supplementary Figure 3A**) and BMI (MD −4.33 kg/m^2^ [95%CI −5.12 to −3.54], 23 studies, 1213 participants) (**Supplementary Figure 3B**) significantly. Similarly, significant TBWL (MD 13.61 [95%CI 11.26 to 15.97], seven studies, 366 participants) (**Supplementary Figure 3C**) and EWL (MD 31.14 [95%CI 21.72 to 40.56], eight studies, 458 participants) (**Supplementary Figure 3D**) were observed following EBMTs.

### 3.11 Adverse Events

Regarding the safety of EBMTs, twenty-one studies provided information about adverse events, but the reporting was inconsistent and fragmented (**Supplementary Table 1**). Common adverse events include nausea, vomiting, abdominal pain, *etc.* The incidence of serious adverse events ranged from 0% to 19% across the individual studies.

### 3.12 Assessment of Risk of Bias

The risk-of-bias tool within CENTRAL was used to assess the risk of bias of RCTs (**Supplementary Table 2**). Two RCTs (32, 33) were evaluated as high risk, and two (21, 43) as unclear. All four RCTs had an unclear risk of bias of outcome. Both studies for AT (32, 33) were at a high risk in the domain of “absence of masking of participants”. The study by Lee and coworkers (21) had an unclear risk of bias for random-sequence generation. We used MINORS criteria to assess the quality of observational studies (**Supplementary Table 3**). The scores were 19 or 20 for comparative studies and ranged from 9 to 13 for noncomparative studies. For the included observational studies, a high risk of bias was found in the domain of “prospective calculation of the study size”, “prospective collection of data”, and “loss to follow-up”. An unclear risk of bias was found for “unbiased assessment of the study end point”. There was unclear and high risk of bias for the domain of “inclusion of consecutive patients”. Assessment of the risk of bias was not done in conference abstracts due to insufficient information.

## 4 Discussion

This meta-analysis explored the potential role of EBMTs for NAFLD by evaluating liver parameters, metabolic parameters, and weight loss. According to our meta-analysis, EBMTs reduced the serum level of ALT, AST, and GGT considerably. Further meta-regression analysis revealed that the magnitude of reduction of the level of liver enzymes was positively correlated with the liver-enzyme level at baseline. Liver steatosis (CAP and HSI), NAS, and insulin resistance improved following EBMTs. With regard to the effects of EBMTs on liver fibrosis, our meta-analysis indicated a significant decline of NFS, but not transient elastography-detected liver stiffness or FIB-4. Moreover, EBMTs had reducing effects on the serum levels of TG and TC as well as body weight.

Recently, Jirapinyo and colleagues (47) carried out a meta-analysis to explore the effects of FDA-approved EBMTs on NAFLD. Significant improvement of liver fibrosis and other NAFLD surrogates (e.g., ALT level, hepatic steatosis, histologic NAS) were observed following EBMTs. In addition, metabolic parameters (insulin resistance and waist circumference) were improved significantly. Based on those results, Jirapinyo and coworkers suggested that EBMTs might ameliorate NAFLD. However, non-FDA-approved EBMTs were not included in their meta-analysis.

For more comprehensive evaluation of the role of EBMTs in NAFLD, we undertook a meta-analysis including FDA-approved EBMTs and non-FDA-approved small intestinal EBMTs such as DJBL and DMR. Our results are mostly consistent with the findings of Jirapinyo and colleagues. Importantly, we found that non-FDA-approved small intestinal EBMTs showed comparable effects with those of FDA-approved devices in terms of improvement in liver parameters.

In our meta-analysis, liver fibrosis was evaluated by non-invasive biomarkers, such as liver stiffness, NFS (48), and FIB-4 (49). EMBTs could decrease NFS significantly, but a significant change in liver stiffness or FIB-4 was not observed. All four studies reporting liver stiffness showed a significant decrease in liver stiffness, which was inconsistent with the results of our meta-analysis. The small study size, diverse disease types, and diverse intervention types may account for this difference. Although these non-invasive biomarkers had high accuracy for detecting clinically significant liver fibrosis, liver biopsy remains the standard for NAFLD diagnosis (50). Two studies have reported the effects of EBMTs on the histologic liver fibrosis. A randomized sham-controlled trial in Singapore evaluated the efficacy of the BioEnterics intragastric balloon (BIB) in ameliorating histology-confirmed NASH (21): a significant change in the fibrosis score was not observed in the BIB group compared with that in the sham group (*P* = 0.303) (21). More recently, an open-labeled prospective study in the USA with 20 NASH patients showed that histologic fibrosis improved in 3/20 patients, remained unchanged in 12/20 patients, and worsened in 5/20 patients, following IGB (9). Future large-scale, randomized controlled clinical trials exploring the effects of different types of EBMTs on histologic fibrosis features are warranted.

When broken down into different types of EBMTs, we found that IGB (the most well-established EBMT available) could improve the level of liver enzymes and histologic NAS significantly, data which are in line with other studies (51, 52). A recent meta-analysis demonstrated that the beneficial effects of swallowable IGB on liver enzymes and insulin resistance were no worse than those of endoscopic IGB (52). As safe and efficacious procedures, the potential utility of IGB for NAFLD has been mentioned in the clinical practice guidelines for NAFLD set by the Asian Pacific Association. Future studies are needed to clarify the long-term efficacy of IGB for NAFLD/NASH patients as well as the safety of IGB in patients with end-stage liver disease.

The potential mechanisms of EBMTs for NAFLD treatment are not clear, but are likely to be related to aspects dependent and independent of weight loss. Insulin resistance is pivotal for NAFLD progression (1). Weight loss could lead to an improvement of insulin resistance in skeletal muscle, thereby improving NAFLD. Weight loss-independent mechanisms differ among different types of EBMTs, in which gut hormones may play a crucial role (53). It has been reported that IGB therapy reduced plasma ghrelin levels (54) and increased levels of sirtuin-1 (55) (a well-known regulator of energy homeostasis and metabolism). Following DJBL implantation, increased fasting and postprandial glucagon-like peptide-1 (GLP-1) levels have been observed (34, 56). GLP-1 can stimulate glucose-dependent insulin secretion, thereby reducing postprandial hyperglycemia. Moreover, alteration of the gut microbiota was observed when DJBL was *in situ*, and returned to baseline levels after removal of the device, which may account for the beneficial effects of DJBL for NAFLD (57).

There was significant heterogeneity among the included studies. We tried to search for possible sources of heterogeneity through subgroup analysis, meta-regression analysis, and sensitivity analysis: only the liver-enzyme levels at baseline were found to be a potential source of heterogeneity. We speculate that two main factors may account for such high heterogeneity: (i) disease types varied from obesity, T2DM, to NAFLD/NASH, and different disease phenotypes may respond differently to a specific therapy; (ii) the diversity of interventions may have led to heterogeneity. Importantly, the magnitude of changes in the levels of liver enzymes was positively corelated with the liver-enzyme level at baseline, indicating that EBMTs could control liver-enzyme levels within the normal range irrespective of NAFLD severity.

Our meta-analysis had five main limitations. First, most of clinical studies in this field are observational studies, which do not allow establishment of firm conclusions. Second, the sample sizes of the included studies were relatively small. Third, most of the included studies focused on the effects of EBMTs in obese cases and/or T2DM patients, and reported changes in liver parameters; only a few studies were done in NAFLD/NASH patients. Further studies are warranted to clarify the safety and efficacy of EBMTs in individuals with different phenotypes of NAFLD. Moreover, the median follow-up time was only 6 months, which did not allow the evaluation of long-term outcomes. Finally, liver histology, the “gold standard” for the diagnosis of NAFLD, was not evaluated in most of the included studies. Therefore, future studies considering the histologic features of NAFLD/NASH are needed.

Despite the limitations stated above, our systematic review and meta-analysis provides the most comprehensive evaluation of the potential role of EBMTs in NAFLD. Eight types of intervention were included in our meta-analysis. Besides, a wide variety of NAFLD biomarkers, including liver histology, non-invasive parameters assessing liver steatosis and fibrosis, metabolic parameters, and weight loss, were evaluated.

## 5 Conclusions

Based on limited evidence, our meta-analysis suggested that EBMTs could ameliorate NAFLD by improving steatosis, NAS, insulin resistance, as well as reducing serum levels of liver enzymes, triglycerides and total cholesterol. EBMTs may bridge a critical gap between less efficacious pharmacologic/behavioral strategies and efficacious but invasive bariatric surgical procedures in NAFLD treatment. Future large-scale trials with rigorous methods are warranted to further clarify the short-term/long-term efficacy and safety of EBMTs for patients with different stages of NAFLD.

## Data Availability Statement

The original contributions presented in the study are included in the article/**Supplementary Material**. Further inquiries can be directed to the corresponding author.

## Author Contributions

Study concept and design, MR and FJ. Written of the protocol, MR. Database searches and study selection, MR and XZ. Risk of bias assessment, MR and MY. Meta-analysis and drafting of the manuscript, MR. Critical revision of the manuscript for important intellectual content, XZ and FJ. Administrative, technical, or material support, YZ, FM, and JY. Study supervision, FJ. All authors have made a significant contribution to this study and have approved the final manuscript.

## Funding

The work was supported in part by a grant from the Key Research and Development Program of Zhejiang Province (2019C03031).

## Conflict of Interest

The authors declare that the research was conducted in the absence of any commercial or financial relationships that could be construed as a potential conflict of interest.

## Publisher’s Note

All claims expressed in this article are solely those of the authors and do not necessarily represent those of their affiliated organizations, or those of the publisher, the editors and the reviewers. Any product that may be evaluated in this article, or claim that may be made by its manufacturer, is not guaranteed or endorsed by the publisher.
