# Supplementary material for: Effects of Bariatric Endoscopy on Non-Alcoholic Fatty Liver Disease: A Comprehensive Systematic Review and Meta-Analysis
Source: Front Endocrinol (Lausanne). 2022 Jun 17;13:931519. doi: 10.3389/fendo.2022.931519 (PMC9247213; doi:10.3389/fendo.2022.931519)
Supplement: Supplementary file 1 [file DataSheet_1.docx]

**Supplementary Appendix 1.** Search strategies

*MEDLINE*

*Nov 2021, 200 results*

("endoscopic bariatric therapy"[Title/Abstract] OR "bariatric endoscopy"[Title/Abstract] OR "intragastric balloon*"[Title/Abstract] OR IGB[Title/Abstract] OR "intra-gastric balloon*"[Title/Abstract] OR "gastric balloon*"[Title/Abstract] OR "stomach balloon*"[Title/Abstract] OR "endoscopic balloon*"[Title/Abstract] OR "intragastric bubble*"[Title/Abstract] OR "gastric bubble*"[Title/Abstract] OR "stomach bubble*"[Title/Abstract] OR "transpyloric shuttle"[Title/Abstract] OR "endoscopic sleeve gastroplasty"[Title/Abstract] OR ESG[Title/Abstract] OR "endoscopic gastric sleeve"[Title/Abstract] OR "endoscopic sleeve"[Title/Abstract] OR "endoscopic gastroplasty"[Title/Abstract] OR "endoscopic gastric plication"[Title/Abstract] OR "endoscopic suturing"[Title/Abstract] OR Overstitch[Title/Abstract] OR "primary obesity surgery endoluminal"[Title/Abstract] OR "primary obesity surgery endolumenal"[Title/Abstract] OR "aspiration therapy"[Title/Abstract] OR AspireAssist[Title/Abstract] OR "duodenal-jejunal bypass"[Title/Abstract] OR "duodenojejunal bypass"[Title/Abstract] OR Endobarrier[Title/Abstract] OR "duodenal mucosal resurfacing"[Title/Abstract] OR Revita[Title/Abstract] OR "incisionless magnetic anastomosis system"[Title/Abstract] OR "incisionless anastomosis system"[Title/Abstract]) AND ("Non-alcoholic Fatty Liver Disease"[Mesh] OR "nonalcoholic fatty liver disease*"[Title/Abstract] OR "non-alcoholic fatty liver disease*"[Title/Abstract] OR NAFLD[Title/Abstract] OR "hepatic steatosis"[Title/Abstract] OR "fatty liver"[Title/Abstract] OR "nonalcoholic steatohepatiti*"[Title/Abstract] OR "non-alcoholic steatohepatiti*"[Title/Abstract] OR liver[Title/Abstract] OR hepatic[Title/Abstract] OR "liver function test"[Title/Abstract] OR aminotransferase[Title/Abstract] OR "alanine aminotransferase"[Title/Abstract] OR ALT[Title/Abstract] OR "aspartate aminotransferase"[Title/Abstract] OR AST[Title/Abstract] OR "alkaline phosphatase"[Title/Abstract] OR ALP[Title/Abstract] OR "gamma-glutamyl transferase"[Title/Abstract] OR GGT[Title/Abstract])

*EMBASE*

*Nov 2021, 493 results*

('endoscopic bariatric therapy':ab,ti OR 'bariatric endoscopy':ab,ti OR 'intragastric balloon*':ab,ti OR IGB:ab,ti OR 'intra-gastric balloon*':ab,ti OR 'gastric balloon*':ab,ti OR 'stomach balloon*':ab,ti OR 'endoscopic balloon*':ab,ti OR 'intragastric bubble*':ab,ti OR 'gastric bubble*':ab,ti OR 'stomach bubble*':ab,ti OR 'transpyloric shuttle':ab,ti OR 'endoscopic sleeve gastroplasty':ab,ti OR ESG:ab,ti OR 'endoscopic gastric sleeve':ab,ti OR 'endoscopic sleeve':ab,ti OR 'endoscopic gastroplasty':ab,ti OR 'endoscopic gastric plication':ab,ti OR 'endoscopic suturing':ab,ti OR Overstitch:ab,ti OR 'primary obesity surgery endoluminal':ab,ti OR 'primary obesity surgery endolumenal':ab,ti OR 'aspiration therapy':ab,ti OR AspireAssist:ab,ti OR 'duodenal-jejunal bypass':ab,ti OR 'duodenojejunal bypass':ab,ti OR Endobarrier:ab,ti OR 'duodenal mucosal resurfacing':ab,ti OR Revita:ab,ti OR 'incisionless magnetic anastomosis system':ab,ti OR 'incisionless anastomosis system':ab,ti) AND ('nonalcoholic fatty liver'/exp OR 'nonalcoholic fatty liver disease*':ab,ti OR 'non-alcoholic fatty liver disease*':ab,ti OR NAFLD:ab,ti OR 'hepatic steatosis':ab,ti OR 'fatty liver':ab,ti OR 'nonalcoholic steatohepatiti*':ab,ti OR 'non-alcoholic steatohepatiti*':ab,ti OR liver:ab,ti OR hepatic:ab,ti OR 'liver function test':ab,ti OR aminotransferase:ab,ti OR 'alanine aminotransferase':ab,ti OR ALT:ab,ti OR 'aspartate aminotransferase':ab,ti OR AST:ab,ti OR 'alkaline phosphatase':ab,ti OR ALP:ab,ti OR 'gamma-glutamyl transferase':ab,ti OR GGT:ab,ti)

*Web of science*

*Nov 2021, 443 results*

TS=(("endoscopic bariatric therapy" OR "bariatric endoscopy" OR "intragastric balloon*" OR IGB OR "intra-gastric balloon*" OR "gastric balloon*" OR "stomach balloon*" OR "endoscopic balloon*" OR "intragastric bubble*" OR "gastric bubble*" OR "stomach bubble*" OR "transpyloric shuttle" OR "endoscopic sleeve gastroplasty" OR ESG OR "endoscopic gastric sleeve" OR "endoscopic sleeve" OR "endoscopic gastroplasty" OR "endoscopic gastric plication" OR "endoscopic suturing" OR Overstitch OR "primary obesity surgery endoluminal" OR "primary obesity surgery endolumenal" OR "aspiration therapy" OR AspireAssist OR "duodenal-jejunal bypass" OR "duodenojejunal bypass" OR Endobarrier OR "duodenal mucosal resurfacing" OR Revita OR "incisionless magnetic anastomosis system" OR "incisionless anastomosis system") AND ("nonalcoholic fatty liver disease*" OR "non-alcoholic fatty liver disease*" OR NAFLD OR "hepatic steatosis" OR "fatty liver" OR "nonalcoholic steatohepatiti*" OR "non-alcoholic steatohepatiti*" OR liver OR hepatic OR "liver function test" OR aminotransferase OR "alanine aminotransferase" OR ALT OR "aspartate aminotransferase" OR AST OR "alkaline phosphatase" OR ALP OR "gamma-glutamyl transferase" OR GGT))

*Cochrane Central Register of Controlled Trials (CENTRAL)*

*Nov 2021, 512 results*

#1 Non-alcoholic Fatty Liver Disease

#2 (endoscopic bariatric therapy):ti,ab,kw OR (bariatric endoscopy):ti,ab,kw

#3 (intragastric balloon*):ti,ab,kw OR (IGB):ti,ab,kw OR (intra-gastric balloon*):ti,ab,kw OR (gastric balloon*):ti,ab,kw OR (stomach balloon*):ti,ab,kw (endoscopic balloon*):ti,ab,kw OR (intragastric bubble*):ti,ab,kw OR (gastric bubble*):ti,ab,kw OR (stomach bubble*):ti,ab,kw

#4 (transpyloric shuttle):ti,ab,kw

#5 (endoscopic sleeve gastroplasty):ti,ab,kw OR (ESG):ti,ab,kw OR (endoscopic gastric sleeve):ti,ab,kw OR (endoscopic sleeve):ti,ab,kw OR (endoscopic gastroplasty):ti,ab,kw (endoscopic gastric plication):ti,ab,kw OR (endoscopic suturing):ti,ab,kw OR (Overstitch):ti,ab,kw

#6 (primary obesity surgery endoluminal):ti,ab,kw OR (primary obesity surgery endolumenal):ti,ab,kw

#7 (aspiration therapy):ti,ab,kw OR (AspireAssist):ti,ab,kw

#8 (duodenal-jejunal bypass):ti,ab,kw OR (duodenal-jejunal bypass):ti,ab,kw OR (Endobarrier):ti,ab,kw

#9 (duodenal mucosal resurfacing):ti,ab,kw OR (Revita):ti,ab,kw

#10 (incisionless magnetic anastomosis system):ti,ab,kw OR (incisionless anastomosis system):ti,ab,kw

#11 (nonalcoholic fatty liver disease*):ti,ab,kw OR (non-alcoholic fatty liver disease*):ti,ab,kw OR (NAFLD):ti,ab,kw OR (hepatic steatosis):ti,ab,kw OR (fatty liver):ti,ab,kw (nonalcoholic steatohepatiti*):ti,ab,kw OR (non-alcoholic steatohepatiti*):ti,ab,kw OR (liver):ti,ab,kw OR (hepatic):ti,ab,kw OR (liver function test):ti,ab,kw (Word variations have been searched)

#12 (aminotransferase):ti,ab,kw OR (alanine aminotransferase):ti,ab,kw OR (ALT):ti,ab,kw OR (aspartate aminotransferase):ti,ab,kw OR (AST):ti,ab,kw OR (alkaline phosphatase):ti,ab,kw OR (ALP):ti,ab,kw OR (gamma-glutamyl transferase):ti,ab,kw OR (GGT):ti,ab,kw

#13 #2 OR #3 OR #4 OR #5 OR #6 OR #7 OR #8 OR #9 OR #10

#14 #1 OR #11 OR #12

#15 #13 AND
