# Supplementary material for: Effects of Bariatric Endoscopy on Non-Alcoholic Fatty Liver Disease: A Comprehensive Systematic Review and Meta-Analysis
Source: Front Endocrinol (Lausanne). 2022 Jun 17;13:931519. doi: 10.3389/fendo.2022.931519 (PMC9247213; doi:10.3389/fendo.2022.931519)
Supplement: Supplementary file 2 [file Image_1.pdf]

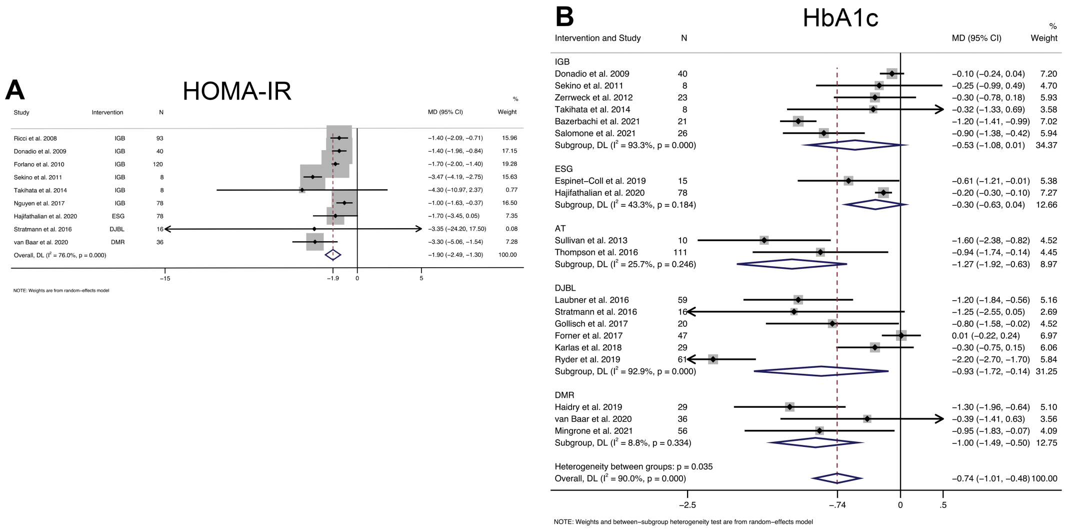

Supplementary Figure 1. Forest plot of changes in (A) Homeostasis Model Assessment of Insulin Resistance (HOMA-IR) and (B) glycated hemoglobin A1c (HbA1c) following bariatric and metabolic endoscopy.
