# Supplementary material for: Effects of Bariatric Endoscopy on Non-Alcoholic Fatty Liver Disease: A Comprehensive Systematic Review and Meta-Analysis
Source: Front Endocrinol (Lausanne). 2022 Jun 17;13:931519. doi: 10.3389/fendo.2022.931519 (PMC9247213; doi:10.3389/fendo.2022.931519)
Supplement: Supplementary file 3 [file Image_2.pdf]

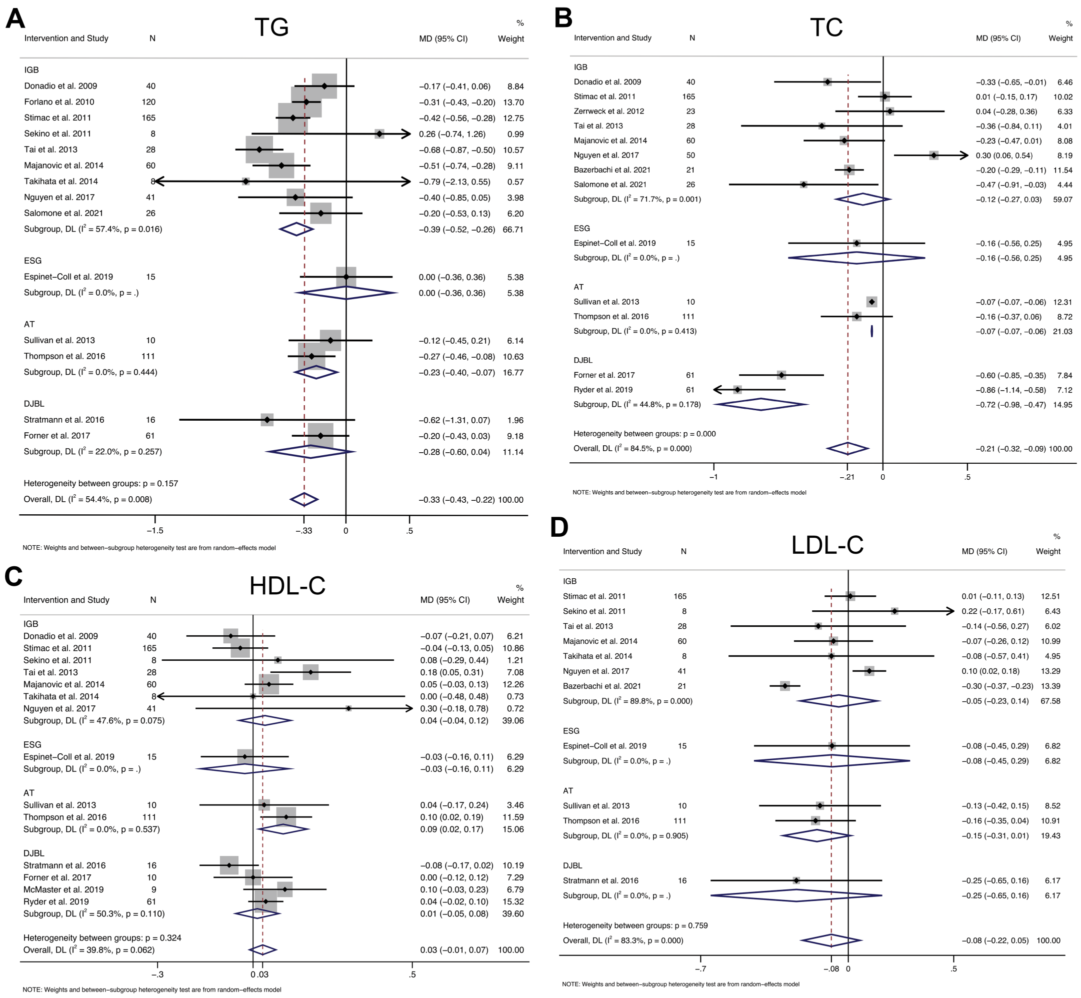

Supplementary Figure 2. Forest plot of change in lipid profiles following bariatric and metabolic endoscopy. (A) serum triglycerides (TG), (B) serum total cholesterol (TC), (C) serum high-density lipoprotein-cholesterol (HDL-C), (D) serum low-density lipoprotein-cholesterol (LDL-C).
