# Supplementary material for: Effects of Bariatric Endoscopy on Non-Alcoholic Fatty Liver Disease: A Comprehensive Systematic Review and Meta-Analysis
Source: Front Endocrinol (Lausanne). 2022 Jun 17;13:931519. doi: 10.3389/fendo.2022.931519 (PMC9247213; doi:10.3389/fendo.2022.931519)
Supplement: Supplementary file 4 [file Image_3.pdf]

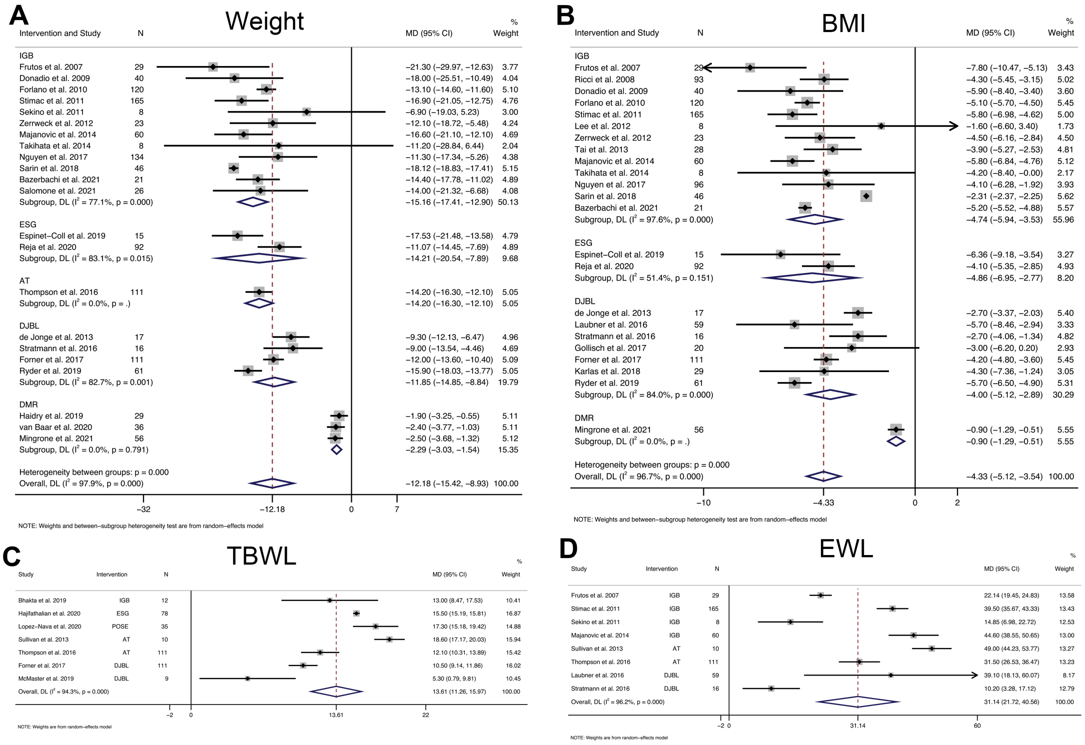

Supplementary Figure 3. Forest plot of weight loss following bariatric and metabolic endoscopy. (A) body weight, (B) body mass index (BMI), (C) total body weight loss (TBWL), (D) excess weight loss (EWL).
