# Supplementary material for: Effects of Bariatric Endoscopy on Non-Alcoholic Fatty Liver Disease: A Comprehensive Systematic Review and Meta-Analysis
Source: Front Endocrinol (Lausanne). 2022 Jun 17;13:931519. doi: 10.3389/fendo.2022.931519 (PMC9247213; doi:10.3389/fendo.2022.931519)
Supplement: Supplementary file 5 [file Table_1.docx]

| **Supplementary Table 1.** Adverse events | | | |
| --- | --- | --- | --- |
| Study | Intervention | Adverse events, n (%) | Serious adverse events, n (%) |
| Frutos et al. 2007 | IGB | Nausea, 27 (93.1%); Vomiting, 25 (86.2%); Rupture of the balloon-inflating device, 1 (3.4%); Post-ingestion epigastralgia, 3 (10.3%); Continuing nausea and vomiting and required removal of the balloon, 2 (6.9%); Intermittent diarrhea with the nausea and epigastralgia, 1 (3.4%) | Hospital admission for intractable vomiting, 1 (3.2%) |
| Ricci et al. 2008 | IGB | NR | NR |
| Donadio et al. 2009 | IGB | Gastroenteric intolerance, 7 (14.9%) | 0 |
| Forlano et al. 2010 | IGB | Early balloon removal, 10 (8%); Mild erosive esophagitis, 2 (1.6%) | 0 |
| Sekino et al. 2011 | IGB | Premature extraction due to psychological intolerance (3, 1.8%) and vanishing of anticipated effect on food intake (1, 0.6%) | 0 |
| Stimac et al. 2011 | IGB | Nausea or vomiting, 4 (50.0%); epigastric pain, 1 (12.5%) | 0 |
| Lee et al. 2012 | IGB | Early removal due to epigastric discomfort and vomiting, 3 (37.5%) | 0 |
| Zerrweck et al. 2012 | IGB | NR | NR |
| Tai et al. 2013 | IGB | Early removal 3 (10.7%) due to panic attack, 1 (3.6%), intractable vomiting, 1 (3.6%) and gastric ulcer with bleeding, 1 (3.6%); Mallory-Weiss syndrome, 1 (3.6%); Erosive esophagitis, 9 (32.1%) | 0 |
| Majanovic et al. 2014 | IGB | NR | NR |
| Takihata et al. 2014 | IGB | Nausea/vomiting, 4 (50.0%); epigastric pain, 1 (12.5%) | 0 |
| Nguyen et al. 2017 | IGB | Nausea and vomiting, 28 (20.7%); Premature removal for intolerance, 14 (10.4%) | Emergency balloon removal for gastrointestinal obstruction, 1 (0.7%) |
| Sarin et al. 2018 | IGB | Nausea, 4 (8.7%); Pain in abdomen, 3 (6.5%); Rupture of the balloon, 1 (2.2%) | 0 |
| ﻿Bhakta et al. 2019 | IGB | NR | NR |
| Bazerbachi et al. 2021 | IGB | NR | NR |
| Salomone et al. 2021 | IGB | Gastroesophageal reflux symptoms were common | 0 |
| Espinet-Coll et al. 2019 | ESG | NR | NR |
| Hajifathalian et al. 2020 | ESG | Mild adverse events (30%); Prolonged pain, 2 (2.2%); Superficial esophageal tear, 1 (1.1%); Peri-gastric leak, 1 (1.1%) | 0 |
| Reja et al. 2020 | ESG | NR | NR |
| Lopez-Nava et al. 2020 | POSE | NR | NR |
| Sullivan et al. 2013 | AT | Abdominal pain, 11 (100%); Peristomal infection, 3 (27.3%); Nausea and/or vomiting, 7 (63.6%); Constipation, 8 (72.7%); Bloating, gas, burping, cramps, 5 (45.5%) | 0 |
| Thompson et al. 2016 | AT | Peristomal granulation tissue, 45 (40.5%); Peristomal irritation, 19 (17.1%); Nausea/vomiting, 19 (17.1%); Intermittent abdominal discomfort, 18 (16.2%); Peristomal inﬂammation, 6 (5.4%) | Severe abdominal pain, 1 (0.9%); Peritonitis, 1 (0.9%); Pre-pyloric ulcer, 1 (0.9%); A-tube replacement, 1 (0.9%) |
| de Jonge et al. 2013 | DJBL | NR | NR |
| Laubner et al. 2016 | DJBL | NR | NR |
| Stratmann et al. 2016 | DJBL | Early explantation due to abdominal pain (1, 6.25%) and migration of the device (2, 12.5%); Gastrointestinal bleeding, 1 (6.25%) | 0 |
| Forner et al. 2017 | DJBL | Mild epigastric pain, 49 (43%); Nausea and vomiting, 17 (15%) | 16 (19%), including life-threatening gastrointestinal hemorrhages (2, 2.5%) and life-threatening liver abscesses (2, 2.5%) |
| Gollisch et al. 2017 | DJBL | Early removal due to abdominal pain (1, 05%), atrial fibrillation (1, 5%), device dislocation (1, 5%) and early satisfaction with the result (1, 59%); Device occlusion, 3 (15%) | 0 |
| Karlas et al. 2018 | DJBL | NR | NR |
| McMaster et al. 2019 | DJBL | NR | NR |
| Ryder et al. 2019 | DJBL | NR | Early removal (10, 16%) due to gastrointestinal haemorrhage (4, 6.4%), liver abscess (2, 3.2%), abdominal abscess (1, 1.6%) and gastrointestinal symptoms (3, 4.8%) |
| ﻿Haidry et al. 2019 | DMR | NR | Duodenal stenosis, 3 (6.8%) |
| van Baar et al. 2020 | DMR | GI symptoms such as diarrhoea, abdominal pain, nausea and oropharyngeal pain, 40 (87.0%); General symptoms such as malaise, fatigue, musculoskeletal pain and rash, 11 (23.9%); Metabolic symptoms such as hypoglycaemia and hyperglycaemia, 3 (6.5%) | 0 |
| Mingrone et al. 2021 | DMR | Abdominal pain, diarrhoea, hyperglycaemia, hypoglycaemia, nasopharyngitis and headache | Mild haematochezia, 1 (1.8%); Jejunal perforation, 1 (1.8%) |
| NR: not reported | | | |
