# Supplementary material for: Effects of Bariatric Endoscopy on Non-Alcoholic Fatty Liver Disease: A Comprehensive Systematic Review and Meta-Analysis
Source: Front Endocrinol (Lausanne). 2022 Jun 17;13:931519. doi: 10.3389/fendo.2022.931519 (PMC9247213; doi:10.3389/fendo.2022.931519)
Supplement: Supplementary file 6 [file Table_2.docx]

| **Supplementary Table 2.** Assessment of risk of bias using the “risk of bias” tool in CENTRAL for randomized controlled trials | | | | | | | | |
| --- | --- | --- | --- | --- | --- | --- | --- | --- |
| Study | Random sequence generation (selection bias) | Allocation concealment (selection bias) | Masking of participants and healthcare providers (performance bias) | Masking of outcome assessment (detection bias) | Incomplete outcome data or excessive loss to follow-up (attrition bias) | Selective reporting (reporting bias) | Other sources of bias | Overall Risk of Bias |
| Lee et al. 2012 | unclear | low | low | unclear | low | low | low | unclear |
| Sullivan et al. 2013 | low | low | high | unclear | low | low | low | high |
| Thompson et al. 2016 | low | low | high | unclear | low | low | low | high |
| Mingrone et al. 2021 | low | low | low | unclear | low | low | low | unclear |
