# Supplementary material for: Effects of Bariatric Endoscopy on Non-Alcoholic Fatty Liver Disease: A Comprehensive Systematic Review and Meta-Analysis
Source: Front Endocrinol (Lausanne). 2022 Jun 17;13:931519. doi: 10.3389/fendo.2022.931519 (PMC9247213; doi:10.3389/fendo.2022.931519)
Supplement: Supplementary file 7 [file Table_3.docx]

| **Supplementary Table 3.** Assessment of risk of bias using the Methodological Index for Non-Randomized Studies (MINORS) criteria for observational studies | | | | | | | | | | | | | |
| --- | --- | --- | --- | --- | --- | --- | --- | --- | --- | --- | --- | --- | --- |
| Study | A Clearly Stated Aim | Inclusion of Consecutive Patients | Prospective Collection of Data | End Points Appropriate to the Aims of the Study | Unbiased Assessment of the Study End Point | Follow-up Period Appropriate to the Aim of the Study | Loss to Follow-up <5% | Prospective Calculation of the Study Size | Adequate Control Group | Contemporary Groups | Baseline Equivalence of Groups | Adequate Stats | Total MINORS |
| Frutos et al. 2007 | 2 | 1 | 2 | 2 | 1 | 2 | 2 | 0 |  |  |  |  | 12 |
| Ricci et al. 2008 | 2 | 2 | 0 | 2 | 1 | 2 | 2 | 0 |  |  |  |  | 11 |
| Donadio et al. 2009 | 2 | 1 | 2 | 2 | 1 | 2 | 2 | 0 |  |  |  |  | 12 |
| Forlano et al. 2010 | 2 | 1 | 2 | 2 | 1 | 2 | 2 | 0 |  |  |  |  | 12 |
| Sekino et al. 2011 | 2 | 1 | 0 | 2 | 1 | 2 | 2 | 0 |  |  |  |  | 10 |
| Stimac et al. 2011 | 2 | 2 | 2 | 2 | 1 | 2 | 2 | 0 |  |  |  |  | 13 |
| Zerrweck et al. 2012 | 2 | 2 | 0 | 2 | 1 | 2 | 2 | 0 | 2 | 2 | 2 | 2 | 19 |
| Tai et al. 2013 | 2 | 2 | 2 | 2 | 1 | 2 | 2 | 0 |  |  |  |  | 13 |
| Majanovic et al. 2014 | 2 | 1 | 2 | 2 | 1 | 2 | 2 | 2 | 2 | 2 | 0 | 2 | 20 |
| Takihata et al. 2014 | 2 | 1 | 2 | 2 | 1 | 2 | 2 | 0 | 2 | 2 | 2 | 2 | 20 |
| Nguyen et al. 2017 | 2 | 1 | 0 | 2 | 1 | 2 | 2 | 0 |  |  |  |  | 10 |
| Bazerbachi et al. 2021 | 2 | 2 | 2 | 2 | 1 | 2 | 2 | 0 |  |  |  |  | 13 |
| Salomone et al. 2021 | 2 | 0 | 0 | 2 | 1 | 2 | 2 | 0 |  |  |  |  | 9 |
| Espinet-Coll et al. 2019 | 2 | 1 | 2 | 2 | 1 | 2 | 2 | 0 | 2 | 2 | 2 | 2 | 20 |
| Hajifathalian et al. 2020 | 2 | 0 | 2 | 2 | 1 | 2 | 0 | 0 |  |  |  |  | 9 |
| de Jonge et al. 2013 | 2 | 1 | 2 | 2 | 1 | 2 | 2 | 0 |  |  |  |  | 12 |
| Stratmann et al. 2016 | 2 | 1 | 2 | 2 | 1 | 2 | 2 | 0 |  |  |  |  | 12 |
| Gollisch et al. 2017 | 2 | 2 | 0 | 2 | 1 | 2 | 2 | 0 |  |  |  |  | 11 |
| Forner et al. 2017 | 2 | 2 | 0 | 2 | 1 | 2 | 2 | 0 |  |  |  |  | 11 |
| Ryder et al. 2019 | 2 | 1 | 2 | 2 | 1 | 2 | 2 | 0 |  |  |  |  | 12 |
| ﻿Haidry et al. 2019 | 2 | 1 | 2 | 2 | 1 | 2 | 2 | 0 |  |  |  |  | 12 |
| van Baar et al. 2020 | 2 | 1 | 2 | 2 | 1 | 2 | 2 | 0 |  |  |  |  | 12 |
| The ideal scores for observational studies are 16 for non-comparative studies and 24 for comparative studies. | | | | | | | | | | | | | |
